# Challenges of diagnosing and managing bronchiectasis in resource-limited settings: a case study

**DOI:** 10.11604/pamj.2019.32.82.18167

**Published:** 2019-02-18

**Authors:** Adebola Adetiloye, Gregory Erhabor, Olayemi Awopeju, Olufemi Adewole, Ebimobowei Onini, Oladapo Adewuya

**Affiliations:** 1 Respiratory Unit, Department of Internal Medicine, Obafemi Awolowo University Teaching Hospitals Complex, Ile-ife, Nigeria; 2 Cardiology Unit, Department of Internal Medicine, Obafemi Awolowo University Teaching Hospitals Complex, Ile-ife, Nigeria

**Keywords:** Bronchiectasis, morbidity, pathogenetic mechanisms

## Abstract

Bronchiectasis, once an orphan disease is now gaining renewed attention as a significant cause of morbidity and mortality. It is a morphologic term used to describe abnormal, irreversibly dilated and thick-walled bronchi, with many etiologies. The management of bronchiectasis can be challenging because its pathogenetic mechanisms is still evolving. Its diagnosis and management is particularly more demanding especially in resource-limited settings like Nigeria because of delayed diagnosis and improper management with devastating consequences, hence this case study.

## Introduction

Bronchiectasis is a morphologic term used to describe abnormal, irreversibly dilated and thick-walled bronchi, with many etiologies [1]. It usually affects the proximal and medium-sized bronchi [2]. The disease was relatively common during the pre-antibiotic era [3], but with improved living conditions, immunization against many childhood respiratory infections and widespread use of antimicrobial agents, it is gradually disappearing in most parts of the highly industrialized societies [4]. The above is however, not reproducible in resource-limited settings, where poverty and high prevalence of respiratory infections still lingers on alongside poor nutrition and inadequate immunization coverage [5]. Consequently, the disease is still encountered in our environment, but diagnosis may be delayed and management challenging [4], leading to huge social and economic loss for patients and their families as many resources are channeled towards combating the disease with undesired results in most cases [6]. Also, information about Bronchiectasis is conspicuously missing from the developing world and in particular, from sub-Saharan Africa, necessitating this report. Adebonojo *et al.* conducted a study in which 70 patients treated for bronchiectasis diagnosed through bronchography at the University College Hospital, Ibadan between 1975 and 1978 and found that there was equal sex prevalence with a wide range of age groups affected. The study also discovered that factors such as malnutrition and chronic anemia adversely influenced the clinical course and prognosis of the disease and emphasized that management was handicapped by inadequate facilities, lack of drugs, illiteracy, poverty, superstitious beliefs and poor environmental hygiene [7].

## Patient and observation

A 43-year-old woman presented to the emergency department with cough, weight loss and progressive difficulty with breathing of 2 months. Cough was insidious in onset, productive of copious thick whitish sputum, which was not foul-smelling but worse early in the morning. There was a history of fever, but no hemoptysis, drenching night sweats or contact with persons with chronic cough. At about the same period, she developed difficulty with breathing which was gradual in onset, provoked by ordinary activities such as walking and doing house chores. Difficulty with breathing progressively worsened, and became present even at rest which made her present at the emergency department. There was associated easy fatigability and orthopnea, but no Paroxysmal Nocturnal Dyspnea. She had bilateral leg and abdominal swelling, but no swelling in other parts of the body and no change in urine volume or frequency. She also complained of unintentional weight loss. Prior to the current bout of symptoms, she has been having recurrent cough for 22 years and had received four courses of anti-tuberculosis medications in the past;1^st^ treatment in 1996, 2^nd^ treatment in 2001, 3^rd^ treatment in 2007 and 4^th^ treatment in 2013. The basis of pulmonary Tuberculosis (PTB) diagnosis could not be ascertained in all cases and she did not complete 6 months of treatment in at least 2 of the courses. There is history of use of firewood for cooking for about 25 years, but no history of cigarette-smoking. She is not diabetic or hypertensive. No history of chronic use of immunosuppressives, exposure to asbestos, sharing of sharps, multiple sexual partners or blood transfusion. She is not a known asthmatic and no history of atopy. No history of recurrent joint pains, skin rash, mucosal sores, recurrent sinusitis or symptoms suggestive of malabsorption. No history of recurrent childhood upper respiratory tract infections. Immunization history could not be ascertained. She does not take alcohol. She is a petty trader and has been divorced for over 5 years due to recurrent ill health and has 4 children. Over the years, she has been patronizing patent medicine dealers, and has been to several hospitals where she had repeated chest x-rays and been on medications including courses of antibiotics with short lived clinical improvement. She also had several courses of antibiotics in the current illness with no improvement prior to presentation. At presentation, she was ill-looking, in respiratory distress, not pale, febrile (38.2°C.), anicteric, cyanosed, not dehydrated, no asterexis, no peripheral lymphadenopathy, had grade 1 finger clubbing and bilateral pitting pedal edema up to the knee. SpO_2_ was 84%, weight was 47kg, and height was 1.65m with BMI of 17.3kg /m^2^.

**Figure 1:**
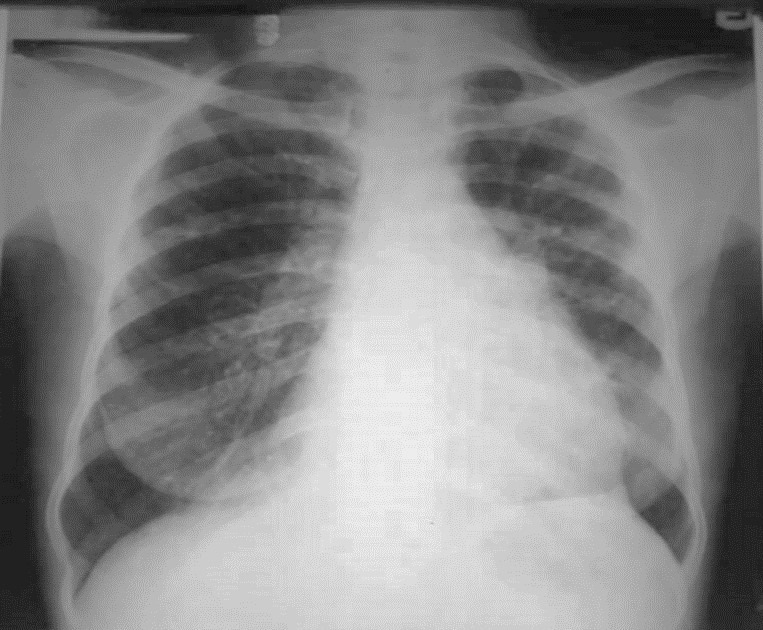
Chest X-ray showing moderate cardiomegaly with mild vascular engorgement with perivascular cuffing

**Figure 2:**
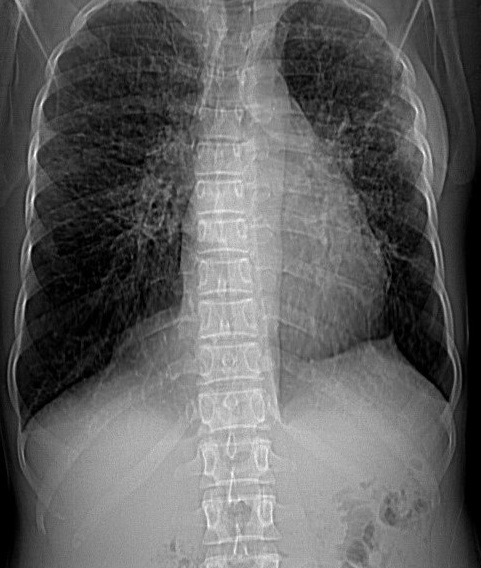
Chest CT scan scannogram reveals severe honeycombing especially in the middle and upper lobes of both lung fields from combination of tramline and bronchial thickening

**Figure 3:**
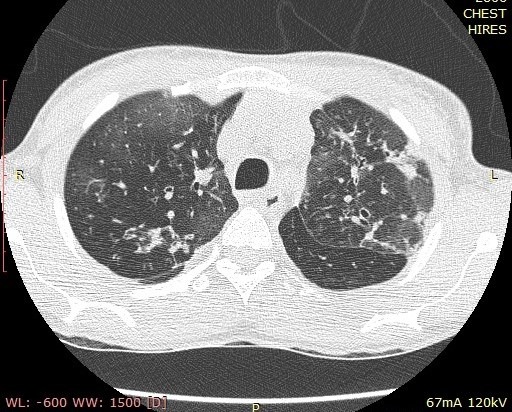
Chest CT scan showing Signet ring appearance in both lung fields in the upper lobes

**Figure 4:**
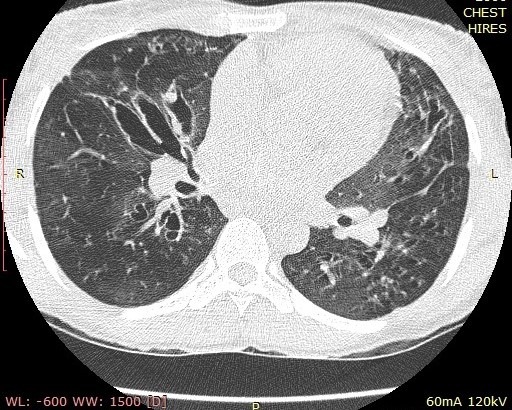
Chest Axial CT scan showing cystic and cylindrical dilatation of the bronchi with bronchial wall thickening centrally, more severe on the left

Chest examinations revealed respiratory rate of 32cpm. Modified medical research council (MMRC) dyspnea scale was 4. Other findings were bibasal coarse crepitations and left lower lobe consolidation. Cardiovascular examination revealed pulse rate of 110 bpm with regular rate and rhythm, Blood pressure 120/70 mmHg, elevated Jugular venous pressure with distended neck veins, Apex beat is displaced laterally with left parasternal heave. She has a third heart sound with loud P2 and pansystolic murmur loudest in the tricuspid area. There was tender hepatomegaly but neurological and musculoskeletal examinations were normal. The most recent Chest X-ray (CXR) done three days prior to presentation showed reticulonodular opacities, cystic lesions especially in the mid to lower zones, moderate cardiomegaly with mild vascular engorgement with perivascular cuffing (Figure 1). Differentials at presentation were PTB complicated by cor pulmonale with superimposed bacterial infection, Post-TB Bronchiectasis, Chronic Obstructive Pulmonary Disease (COPD), Congestive heart failure secondary to valvular heart disease and Interstitial lung disease. Patient was admitted and various investigations were ordered. She was nursed in cardiac position and the following treatments were commenced; Intranasal oxygen at 5L/min, intravenous (IV) Frusemide 60mg daily, Tab Spironolactone 25mg daily, IV Augmentin 1.2g 12 hourly, Tab clarithromycin 500mg bd and chest physiotherapy. Chest Computerized Tomography (CT) scan revealed lung fields showing mosaic pattern with patchy areas of ground glass attenuation. Multiple poorly defined nodules of varying sizes and shapes are seen in a random distribution in all the lung segments with upper lobe predominance. Conferencing nodules are seen in the apical segment of the right lung, apicoposterior segement of left lung with extension into the oblique fissure and lateral aspect of superior lingular segment. Generalized cylindrical, varicoid and cystic bronchiectatic changes with signet ring sign is seen in both lung fields but worse at the right middle lobe and left lingular segments. There is thickening of the intralobular septae and the walls of the dilated bronchi. The impression was Bronchiectasis with mosaic perfusion and randomly distributed pulmonary nodules most likely to Post infectious (Figure 2, Figure 3, Figure 4).

### Results of other investigations are as follows

Sputum studies microscopy, culture and sensitivity yielded growth of upper respiratory flora; AFB and GeneXpert were negative for MTB. Blood culture yielded no growth. Fingerstick glucose was 6.6mmol/L at presentation. Basal metabolic panel was within normal limit (HCO_3_: 28 mmol/L, Na:128 mmol/L, K: 3.5 mmol/L, Urea: 2.4 mmol/L, Cr:81 umol/L). FBC revealed PCV: 42%; WBC: 12,000 cells/mm^3^, Platelets: 372,000 cells/mm^3^, Neutrophil: 84%, Lymphocytes: 44%, Eosinophils: 02%. ESR was 33mm/hr (westergren method). ECG showed Sinus tachycardia and Right axis deviation. Echocardiography revealed Right heart disease. Anti-nuclear antibody and HIV screening were both negative. Serum proteins were within normal limits. Spirometry revealed obstructive pattern (Table 1). Based on the Chest CT scan findings and result of other investigations, a diagnosis of Bronchiectasis probably post-PTB or secondary to poorly treated pneumonia, with infective exacerbation, complicated by corpulmonale was made. Nebulized salbutamol was added to her current treatment regimen. After 6 days on admission, patients condition deteriorated evidenced by persistent fever, respiratory distress, confusion and hypoxia (SpO_2_ 82%) and was considered for Intensive care unit admission, bronchoscopic sampling of the lower respiratory tract, while IV Augmentin was changed to IV Cefepim. The relatives however, objected to further treatment due to financial constraint and was subsequently discharged against medical advice. She was also advised to get multivalent pneumococcal polyvalent vaccine at the nearest primary health care center upon discharge.

**Table 1:** Spirometry showing obstructive pattern

Parameter	Pre-bronchodilator	Percentage of predicted (%)	Post-bronchodilator	Percentage of predicted (%)	Percentage of change (%)
FEV1(L)	0.77	38	0.82	35	+6
FVC (L)	1.67	60	1.51	55	-10
FEV1/FVC	46.1	55	54.3	65	+18
PEF(L/s)	1.26	21	1.68	28	+33

## Discussion

Bronchiectasis is a highly devastating illness with frequent exacerbations especially in severe disease [8]. This can place a huge burden on the health system as patients utilize many health care resources including numerous clinic visits, hospitalizations, diagnostic imaging and parenteral antibiotics [9]. In resource-limited settings like Nigeria, diagnosis and management of bronchiectasis have been hampered by paucity of basic resources for effective evidence-based case management together with other factors. Patients generally have poor health-seeking behavior, which usually leads to delay in diagnosis and treatment. As a result, they present with advanced disease leaving the pulmonologist with limited options for management. Sadly, this is further compounded by problems posed by medical quackery, influence of religion and culture, poverty, inadequate referral system and poor drug adherence. The above constitute general challenges encountered in management of any disease condition in resource-limited settings to which bronchiectasis is not alien. More specifically, management of Bronchiectasis in resource-limited settings has been challenged by the existence of clinical overlap between bronchiectasis and other lung diseases. It therefore requires the use of standard diagnostic modalities such as High-Resolution Computed Tomography (HRCT) for prompt diagnosis. The lack of formidable multidisciplinary team in patient care is another challenge. The management of bronchiectasis requires an all-encompassing team that includes the pulmonologist, nutritionist, infectious disease specialist, psychologist, physiotherapist and adequate nursing staff as well as the surgeon [10]. It follows therefore, that availability of sufficient human resources is also key in the proper management of bronchiectasis patients. Our patient did not receive such care due to paucity of staff coupled with poor socio-economic status. Moreover, there was conspicuous lack of physiotherapy equipment such as mechanical valve devices and therapeutic vests which serve integral part of the management of patients with Bronchiectasis. These facilities are not only not easily accessible in Nigeria, but also come with high costs, making it particularly difficult for patients in our setting to benefit from such services. It becomes imperative, therefore, that the best way to cut down on the existence of bronchiectasis as a disease entity in resource-limited settings is to adopt disease prevention as the watchword. This can be achieved at three levels of prevention, vis-a-viz; primary, secondary and tertiary [11]. Primary prevention involves health education and avoidance of environmental risk factors for lung infection. Such measures include avoidance of direct and indirect tobacco smoking, ensuring childhood immunization especially measles and pertussis, avoidance of indoor and outdoor air pollution and improvement of living conditions.

Other primary measures to prevent the development of post infectious bronchiectasis include prompt diagnosis and treatment of chest infections, which can be fostered by early presentation. Necrotizing lung infections, including fulminant and inadequately treated pneumonia caused by Klebsiella, Staphylococcus and some serotypes of Streptococcus could also result in bronchiectasis. In areas with more deprived socioeconomic and health-care provision, bronchiectasis is likely a common complication of such infections [12]. Improving access of patients to essential drugs, medication adherence and regular training of health care personnel to make diagnosis and treat pneumonia appropriately, are important preventive measures. Appropriate antituberculous therapy cannot be overemphasized as tuberculosis accounts for a significant proportion of bronchiectasis in resource-limited environments like Nigeria [12]. Secondary preventive measures to deter progression of disease in patient who have already developed bronchiectasis is early diagnosis, and this requires high index of suspicion and availability of diagnostic equipment. Plain CXR finding of “tram-lines” reflecting underlying thickened and dilated bronchi is often unreliable for diagnosis of bronchiectasis, which accounted for one of the reasons the diagnosis was delayed in this patient [12]. HRCT is the current working gold standard for diagnosing bronchiectasis, because it has a very high sensitivity and specificity. The distribution (apical versus basal and central versus peripheral) and concomitant findings, such as nodules, cavities, and/or lymphadenopathy, that can assist in narrowing the differential diagnosis [13]. HRCT is considered expensive in Nigeria due to poverty and lack of health insurance. In addition, only a few centers are equipped with this facility making ready availability a factor impeding prompt diagnosis. However patients need to be educated on the need for early diagnosis and encouraged to do appropriate investigations to enable early detection and management. Our patient had repeated treatments for pulmonary tuberculosis and several courses of antibiotics before a definitive diagnosis with HRCT was made, at which point the disease was already advanced with ensuing complications. Following diagnosis, treating the underlying cause of bronchiectasis can also slow or prevent its progression. However, the etiology may not be apparent in about 50% of cases despite intensive evaluation. Furthermore, only about 15% of patients have causative factors amenable to specific treatment according to a study [14]. The etiology of bronchiectasis in the index patient is not precisely known but based on the distribution of HRCT findings and epidemiologically, a post infectious cause is highly probable. Investigations such as screening for alpha-1 antitrypsin deficiency, cystic fibrosis, humoral immune defects, Aspergillus precipitins and other autoimmune markers were not done in this patient because of cost and availability. However, our patient had no features suggestive of some of these diseases such as malabsorption, rashes, arthralgia, liver disease, recurrent sinusitis, childhood infections or gastrointestinal infections.

Research has shown three important pathogenic and interactive components in bronchiectasis, namely infection, inflammation and enzymatic components of pathogenesis. These components cause chronic and self-perpetuating destruction to the airways, leading to further deterioration in bronchiectasis [15]. In contrast to healthy non-smokers, the airway in bronchiectasis is frequently colonized with potentially pathogenic microorganisms. The most commonly found microorganism was H. influenza (55%), followed by Pseudomonas species (26%) and Streptococcus pneumonia (12%). Other important pathogens include Moraxella catarrhalis, Aspergillus and Mycobacterium avium complex (MAC). Staphylococcus aureus relatively uncommon in non-cystic fibrosis bronchiectasis (NCFB) [16-18]. Studies suggest that loss of diversity, with dominance of one or a few species, is associated with worse lung function and more exacerbations. In addition, Pseudomonas aeruginosa dominance has been associated with worse lung function and more exacerbations [19]. Since progression of the disease is linked to impaired mucus clearance, airway colonization by bacteria, airway inflammation as well as airway structural damage, the therapeutic goal should be to halt or reverse these processes and break the cycle [20]. Just like other chronic lung diseases, patients with bronchiectasis should be encouraged to stop smoking and receive vaccination against influenza and pneumococcal disease. The benefits of management of treatable of identifiable causes cannot be underscored although many patients such as the index case had no readily identifiable cause [21]. The FACED score is composed of FEV1, age, chronic colonization with Pseudomonas, radiographic extent of disease, and degree of dyspnea demonstrate a strong ability to predict mortality 4-5years from the time of diagnosis. These scores allow for the division of bronchiectasis into three severity classes: mild, moderate, or severe [22]. The index patient had severe bronchiectasis according to this score and should have received a more intensive management. Antibiotics are the cornerstone for the management of bronchiectasis. They are used to treat acute exacerbations, to prevent exacerbations, or to reduce the bacterial burden. Antibiotic choices should be guided by most likely offending organisms epidemiologically, and where possible, by previous sputum microbiology and sputum surveillance which is not a common practice in our setting, leading to ineffective antibiotic choices. In adults, bronchoscopy and bronchoscopic sampling of the lower respiratory tract does not have a place in the routine investigation of patients with bronchiectasis but for patients in whom serial testing of sputum does not yield microbiological information and who are not responding well to treatment, bronchoscopic sampling of lower respiratory tract secretions may be indicated [23,24].

Patients with bronchiectasis often require higher and prolonged doses of antibiotics during infective exacerbation to effective tackle the problem of biofilm formation by bacteria which had been implicated in chronic disease like bronchiectasis. The most important characteristic of bacterial biofilms, is a decreased susceptibility to antimicrobial agent. This decreased susceptibility has two aspects, tolerance and resistance. Tolerance means that bacteria are not killed, although they are unable to grow in the presence of the drug, whereas resistance allows bacteria to grow in the presence of antibiotics [25]. The appropriate length of treatment for exacerbations is not known, but consensus guidelines recommend 14 days of treatment with antibiotic therapy guided by previous sputum microbiology [24]. The index patient received suboptimal doses and sometimes inappropriate antibiotics with poor adherence during periods of exacerbations of chest infections; these could have contributed to bacterial resistance and progression of her disease. Whether prophylactic antibiotic therapy is necessary remains an unresolved question. Patients who experience frequent exacerbations may benefit from a maintenance regimen, but the evidence for this approach is fairly weak. British Thoracic Society (BTS) guidelines recommend consideration of long term oral antibiotics for patients with ≥ 3 exacerbations a year or those chronically colonized with Pseudomonas aeruginosa. Strategies for prophylaxis with low-dose antibiotics range from daily to 1 week of each month [24]. Maintenance antibiotics has been shown to prevent detrimental infections and decrease hospitalizations in people with bronchiectasis according to a report [26].

There has been increased interest in macrolides for the treatment of NCFB because of their anti-inflammatory properties and ability to decrease mucus production. Illustrating this, Liu and colleagues showed that in patients with NCFB, 6 months of therapy with roxithromycin significantly reduced various inflammatory markers [27]. Another study demonstrated that low-dose clarithromycin decreased levels of CD4+IL-17+ cells in peripheral blood and IL-17 levels in exhaled breath condensate in patients with NCFB [28]. These studies further add to the growing evidence that macrolides may modulate inflammatory patterns in NCFB. The index patient could have benefitted from maintenance antimicrobial therapy giving the severity of her disease. The role of prophylactic inhaled antibiotics in NCFB is evolving with the aim of delivering the drug directly into airway to improve antibacterial efficacy and to reduce systemic side effects. Antibiotics that have been tried include the aminoglycosides (nebulized gentamicin/tobramycin), colistin, ciprofloxacin, and aztreonam; they have collectively demonstrated a reduction in sputum bacterial load [29]. These approach to management may not be feasible in our environment due to unavailability of these agents and high cost of procurement. Although long-term outcomes are lacking, it has been demonstrated that high doses of bromhexine coupled with antibiotics improved mucus clearance compared with placebo. They found that erdosteine when combined with physiotherapy over a 15-day period improved spirometry and sputum purulence compared with physiotherapy alone [30]. Therefore, patients need to be routinely referred for standardized chest physiotherapy to improve their quality of life amongst other benefits. Inhaled hyperosmolar agents like hypertonic saline and mannitol, mechanistically draw fluid and mucus into the airway, enhancing clearance. Small trials have shown equivocal benefit with hypertonic saline on clinical outcomes, such as exacerbation rate, quality of life, and lung function, and larger studies are required [31]. Mannitol is readily available and affordable in our environment and its efficacy or lack thereof can be documented if used in our patients.

Patients with localized bronchiectasis with persistent symptoms despite maximal therapy, or recurrent infections with resistant pathogens may benefit from surgical removal of affected segments or lobes. A study of patients who underwent surgical treatment for NCFB has highlighted positive outcomes even in patients with nonlocalized bronchiectasis. In this study, the most common procedure was segmentectomy and lobectomy [32]. If our index patient was diagnosed earlier and had localized disease, she may had benefitted from surgical treatment which could have prevented other parts of the lungs from been infected. A review by Goyal and Chang compared the value of combined inhaled corticosteroid (ICS) and long-acting ß2-agonist versus high-dose ICS monotherapy and found that the combination inhaler improved subjective dyspnea and increased cough-free days compared with high-dose ICS alone [33]. However, a study on the complications of inhaler use found that there was a significantly increased risk of hemoptysis 53 days after initiation of ICS/long-acting ß2-agonist or short-acting ß2-agonist [34]. However, the routine use of bronchodilators has the added potential advantage of the stimulation of mucociliary clearance, which is associated with the use of ß-adrenergic agents. Both aerosolized ß2-agonist therapy and aerosolized anticholinergic therapy should be tried when there is evidence of reversible airway obstruction [23]. Our index patient had obstructive pattern on spirometry but due to non-adherence to ICS and ß2-agonist, a clear improvement in symptoms cannot be ascertained. Long-term oxygen therapy (LTOT), adequate family and social support as well as effective palliative are tertiary preventive modalities to minimize physical and psychosocial complications that could arise as a result of bronchiectasis [31]. Bronchial artery embolisation and/or surgery is first-line therapy for the management of massive hemoptysis which is not uncommon in patient with bronchiectasis and a cause of mortality in some patients [24]. Most Tertiary hospitals don't have access to this technique in which case, therapeutic bronchoscopic measures, endotracheal intubation and other modalities have led to successful treatments in some cases. Weitzenblum mentioned that bronchiectasis is one of the obstructive diseases that can lead to the development of corpulmonale [35]. Our patient had clear features of corpulmonale as previously mentioned. Leong *et al.* and Fishman described pulmonary hypertension as the common link between lung dysfunction and the heart in corpulmonale [36, 37]. The traditional view of the development of right heart failure (RHF) has an indicator of poor prognosis in chronic respiratory patients [38], has been interrogated. However, it is now assented that a prolonged survival (≥ 10 years) can be discerned after the first occurrence of peripheral edema. This is because; the prevalence of clinical RHF has greatly decreased with the application of long term oxygen therapy (LTOT), with a resulting improvement in prognosis [35].

Double-lung transplantation in patient with widespread severe disease with complications, has been successful in CF patients. According to a report, more than 1,000 CF patients and 200 non-CF bronchiectasis patients who have undergone lung transplantation, with a 1-year survival rate of 72% and a 4-year survival rate of 49% in patients with CF [23]. Double lung transplantation may also reverse right ventricular failure in some patients [39]. Lung transplantation is presently not performed in Nigeria and majority of our patients cannot afford medical tourism which is also fraught with long waiting list for organ donors. Overall, the above measures require more research and clinical effort to change the attitude of patients and their care-givers by proper education, improved clinical practice, as well as the governments political will to better equip and fund the health sector. The understanding of bronchiectasis is evolving and this may revolutionize the management of this orphan disease that is now gaining more attention because of its devastating effects. Development of novel therapeutic agents targeted at pathogenetic factors such as neutrophil elastase inhibitors, bacterial biofilm dispersers, statins, chemokine receptor 2 (CXCR2) antagonists, new anti-pseudomonal compound based on the antimicrobial peptide protegrin, are welcome developments, given the complexities and drawbacks to the current treatment of bronchiectasis [21,40].

## Conclusion

Bronchiectasis was initially thought to be an orphan disease, but is increasingly gaining widespread recognition due to a current rise in its incidence. It is still a source of morbidity and mortality in resource-limited settings due to a variety of challenges in its diagnosis and management. The linchpins in the prevention and management of bronchiectasis are, the timely institution of appropriate antimicrobials to treat lung infections, high index of suspicion and prompt diagnosis and, a more aggressive management of infections in diagnosed cases to retard the progression of the disease.

## Competing interests

The authors declare no competing interest.
